# MiRNA-3Age: a microRNA-based biological age model and its modulation by lifestyle and nutrition

**DOI:** 10.3389/fnut.2025.1659730

**Published:** 2025-11-20

**Authors:** Jana Schneider, Clara Preyer, Marie Steil, Maruan Biazid, Angelika Pointner, Alexander G. Haslberger, Berit Hippe

**Affiliations:** 1 Department of Nutritional Sciences, University of Vienna, Vienna, Austria; 2 HealthBioCare GmbH, Vienna, Austria

**Keywords:** epigenetics, microRNA, aging, nutrition, lifestyle, healthspan, aging clock, biological age

## Abstract

**Introduction:**

The extension of human longevity has intensified the search for biomarkers that capture not only chronological age but also biological aging and functional healthspan. Among molecular candidates, microRNAs (miRNAs) have emerged as promising regulators and indicators of aging-related processes. In this pilot study, we explored whether selected circulating miRNAs could serve as potential biomarkers of biological age and lifestyle-associated aging dynamics.

**Methods:**

Based on current literature, we focused on three miRNAs—miR-24, miR-21, and miR-155—previously linked to inflammation, senescence, and metabolic regulation. Capillary blood samples from a heterogeneous adult cohort were analyzed using quantitative PCR. ΔCt values were integrated into a composite “miRNA-3Age” model through multivariate regression analysis to estimate biological age. Associations between lifestyle variables (diet, exercise, stress, and smoking) and miRNA-based biological age were examined.

**Results:**

The miRNA-3Age model predicted biological age with moderate correlation to chronological age and revealed variability consistent with individual health profiles. Participants with favorable lifestyle factors (e.g., frequent consumption of fish, whole grains, and green tea; regular exercise) tended to exhibit lower miRNA-3Age estimates, whereas stress and smoking were associated with higher predicted biological age.

**Discussion:**

Our exploratory data suggest that integrating multiple miRNA signals may enhance the sensitivity of biological age estimation compared to single-biomarker approaches. However, variability within the small sample highlights the need for larger, longitudinal datasets to confirm predictive validity and to disentangle causal links between lifestyle, miRNA expression, and aging biology.

**Conclusion:**

This pilot study supports the feasibility of miRNA-based biological age modeling and identifies miR-24, miR-21, and miR-155 as promising components of a composite biomarker framework. The miRNA-3Age model provides a preliminary step toward a scalable, lifestyle-sensitive aging metric that warrants validation in diverse populations.

## Introduction

Aging is a multifactorial natural process accompanied by physical and mental decline with an increasing risk of diseases such as cancer, diabetes, and cardiovascular diseases (1–3). This process is often associated with elevated oxidative stress levels, which lead to enhanced expression of pro-inflammatory factors including cytokines, chemokines, and growth factors, resulting in a persistent pro-inflammatory state in various cell types. This condition, termed “inflamm-aging,” is primarily caused by permanent activation of immune cells and increasing accumulation of senescent cells (4, 5).

The Hallmarks of Aging describe fundamental processes that contribute to aging in general and may potentially be modulated. These include accumulation of DNA mutations, epigenetic alterations, cellular senescence, and oxidative stress, among other key mechanisms (1, 3, 6).

As hygiene standards, medical interventions, and lifestyle factors have improved over the years, life span has increased, leading to a demographic shift toward an elderly population (7, 8), healthspan, the time spent living healthy and disease-free, has not extended proportionally (8). It is proposed that by slowing the aging process, healthspan can be increased through targeted health interventions (2). Among the Hallmarks of Aging, epigenetic alterations are potentially reversible and therefore represent an effective target for extending healthspan through anti-aging treatments (1).

The years alive since birth of an individual are referred to as chronological age (CA). Although it is a reliable risk factor for age-related diseases and mortality, it provides an imperfect insight into the aging process, as it is not always accurately linked with actual aging status (6, 9, 10). This limitation has led to increased attention on biological age, which describes the overall health condition of an individual in comparison to their chronological age (6).

Beyond biological age, researchers aim to develop reliable biomarkers of aging that meet specific criteria (3, 9, 11). This research led Horvath to describe a physiological age estimator called DNAm age (Epigenetic clock). In the epigenetic field, two omics-based biomarkers are predominant: DNA methylation age (DNAm age) and telomere length (12).

Recent transcriptomic approaches focus on miRNAs, non-coding RNAs that regulate gene expression post-transcriptionally. As miRNAs are involved in pathways that influence aging, including cellular senescence, inflammation, and DNA repair, they may provide multifaceted insights into aging processes (6).

MicroRNAs (miRNAs) are small single-stranded non-coding RNAs with a length of approximately 21–23 nucleotides that influence gene expression by binding to the 3′ untranslated region (UTR) of target mRNAs (13). About 2,400 miRNAs are identified in human cells, tissues, and organs, where they have been linked to the aging process and age-related diseases (6, 14–16).

Previous research has demonstrated that many miRNAs regulate inflamm-aging through interactions with the nuclear factor κB (NF-κB) signaling pathway or Toll-like receptors (TLRs). Notably, miR-155, miR-21, and miR-146 are co-induced after TLR signaling activation (4, 5). MicroRNAs also play a key role in regulating cellular senescence, particularly stress-induced senescence, by binding to complementary sequences in target mRNAs' 3′ UTR, thereby silencing gene expression (17).

MiR-155 is a key regulator of the immune system predominantly expressed in organs such as the liver, spleen, and thymus. It plays a vital role in immune cell development and controls inflammatory responses in the body. By influencing proinflammatory molecules, it contributes to the body's defense against infections and leads to upregulation in the bone marrow, promoting reactive oxygen species (ROS) production (18, 19). Increased ROS production is associated with accelerated aging, aligning with findings from Sredni et al. showing that miR-155 expression increases in adult women during aging (20).

The overexpression of miR-155 in aging inhibits protective genes, leading to excessive ROS accumulation, linking miR-155 to oxidative stress and inflammation (17, 19). Conversely, lower miR-155 levels can increase TP53INP1, a marker of p53-driven growth arrest. By targeting FOXO3 and HIF-1α, miR-155 also regulates ROS levels, further impacting cellular aging (17, 21).

MiR-21 serves as a biomarker of healthy aging, with levels increasing over time but decreasing in individuals over 80 years and centenarians, suggesting lower miR-21 levels may support longevity. This pattern positions miR-21 as both a marker of aging and a potential target for anti-aging interventions, connecting cellular senescence and inflamm-aging as two key effects of aging (17, 22). Its expression is associated with various age-related diseases, including Alzheimer's, Parkinson's, and type 2 diabetes (23).

MiR-21 is enriched in extracellular vesicles from senescent endothelial cells, facilitating the spread of senescence signals to neighboring cells. It influences epigenetic changes for genomic integrity by targeting DNMT1 and SIRT1, while also affecting antioxidant defense genes like SOD2 and activating ROS-producing enzymes such as NOX4, contributing to oxidative stress and cellular senescence (17, 24).

In inflammatory regulation, miR-21 affects the NF-κB and NLRP3 pathways by inhibiting or promoting these pathways. Mir-21 is functioning as an anti-inflammatory agent by downregulating IRAK and MyD88 in the TLR signaling pathway. In aging organisms with reduced TGF-β signaling, increased miR-21 expression correlates with higher TNF-α and interferon-γ levels, activating the NF-κB pathway as an agonist for TLRs and endogenous ligand of TLR8, triggering inflammatory cytokine release (4, 5, 23, 25).

MicroRNA-24 functions as a crucial regulator in cellular aging, targeting key senescence-related genes including MKK4, p16INK4a, E2F2, and H2AX involved in cell cycle progression and DNA repair (26). It was identified as a novel aging marker in salivary exosomes from young and old participants (13).

MiR-24 regulates the p16 pathway by binding to two sites on its mRNA (coding region and 3′ UTR), preventing p16 activation which inhibits cell proliferation. As cells age, decreasing miR-24 levels lead to p16 accumulation, promoting cellular senescence, establishing an inverse relationship of miR-24 and p16 contributes to senescence in the aging process (16, 27).

Through modulation of H2AX expression and enhancement of oxidative stress-induced senescence via increased p53 levels, miR-24 impacts DNA damage response and may accelerate aging by promoting oxidative stress and premature senescence (26, 28). Additionally, miR-24 targets genes in the MAPK signaling pathway, regulating inflammatory cytokine and chemokine production, thus contributing to age-related changes in immune function and inflammation with increasing miR-24 expression levels (13).

Diet plays a crucial role in regulating physiological processes, thereby impacting various pathological conditions. MicroRNAs have emerged as potential mediators in the complex relationship between dietary factors and health outcomes (29).

Recent investigations suggest that specific dietary components, including vitamins, polyphenols, fatty acids, and particular dietary patterns, can influence metabolic pathways through the modulation of endogenous miRNA expression and function (30). These interactions constitute an important mechanism by which nutrition may exert its effects on cellular processes and overall health.

Beyond endogenous miRNA modulation, dietary miRNAs present in food have been proposed to enter the circulatory system upon digestion, potentially influencing gene regulation in the host (30).

Bioactive compounds have demonstrated significant capacity to regulate miRNA expressions, thereby affecting key cellular processes. Curcumin, for instance, down-regulates oncogenic miR-21 leading to tumor suppression and enhanced apoptosis. Resveratrol modulates miR-21, influencing pathways involved in cell proliferation and inflammation (31).

In addition to miR-21, several naturally occurring dietary flavonoids and polyphenols exert potent anti-inflammatory effects by targeting miR-155, a key regulator of immune responses. Quercetin, abundant in apples, onions, tea, and red wine, reduces the expression of proinflammatory cytokines by downregulating miR-155 and inhibiting the NF-κB signaling pathway. Similarly, resveratrol decreases miR-155 levels and suppresses inflammatory mediators, particularly in the context of metabolic disorders. Apigenin, found in chamomile tea, celery, and parsley, inhibits LPS-induced miR-155 expression by suppressing NF-κB activity and upregulating anti-inflammatory genes such as FOXO3a and SMAD2, leading to reduced TNF-α release. Curcumin also downregulates miR-155 by inhibiting the PI3K/AKT signaling pathway, thereby enhancing its overall anti-inflammatory properties (32).

These molecular interactions illustrate how nutrients and dietary components can exert biological effects through miRNA-dependent mechanisms.

Beyond endogenous miRNA modulation, an emerging hypothesis suggests that dietary miRNAs present in food may enter the circulatory system upon digestion, potentially influencing gene regulation in the host organism (30). This concept, while still debated, suggests another layer of interaction between diet and genetic regulation.

Dietary molecules also influence broader epigenetic mechanisms, including DNA methylation, histone modifications, and non-coding RNA interactions, further contributing to comprehensive gene regulation. Natural compounds (NCs) modulate miRNA expression with dual potential effects. They can exert protective functions by reducing inflammation and regulating immune responses. Conversely, certain NCs may contribute to disease by up-regulating inflammatory and oncogenic miRNAs, thereby promoting pathological processes such as chronic inflammation and tumor progression (29).

While *in vitro* and *in vivo* studies have demonstrated numerous mechanisms by which nutrients affect miRNA expression, human data on dietary miRNA modulation remains limited. This knowledge gap necessitates further research, particularly well-designed human studies, to clarify the full extent and physiological relevance of these diet-miRNA interactions (29).

Building upon established relationships between miRNAs, health status, and nutritional influences, this study investigates specific miRNAs associated with chronological aging to develop a novel miRNA-based age estimation model. This functional estimator complements existing biological age calculators by providing additional insights into epigenetic aspects of aging. We then apply this miRNA-based age estimator to identify and characterize how various lifestyle factors and nutritional patterns influence biological aging. Through this approach, we aim to explain potential mechanisms by which dietary interventions may modulate aging processes at the molecular level.

## Materials and methods

### Cohort recruitment

The study cohort, consisting of 1,151 subjects, was recruited between 2015 and 2023 through an Austrian company specializing in personalized nutrition. Data collection included dietary information, lifestyle factors, medical history, and behavioral characteristics, obtained through self-completed questionnaires. Additionally, whole blood samples were collected from each participant for analysis.

To develop a broadly applicable aging marker for the general population, several exclusion criteria were established. These exclusion criteria encompassed individuals under 18 years of age and participants with specific medical conditions. The excluded conditions included arthritis, high blood pressure, diabetes mellitus type 1 and 2, gout, thyroid dysfunction and inflammatory gastrointestinal diseases.

After applying these criteria, 539 participants qualified for the study, forming a “healthy” baseline population free from the specified conditions.

### MicroRNA selection

Based on literature review, three microRNAs previously associated with aging processes were selected for analysis: miR-21, miR-24, and miR-155. MicroRNA-93 was used as a housekeeping reference for normalization.

### RNA extraction and qPCR

Dried capillary blood samples were collected on Whatman protein saver cards using 18G safety lancets. DNA and RNA extraction was performed using a magnetic bead-based method over two days. On day one, blood card punches were incubated overnight in protease buffer using a ThermoMixer. On day two, extraction was completed using the KingFisher™ Duo Prime Magnetic Particle Processor and MagMax FFPE DNA/RNA Ultra kit (ThermoScientific™).

The extracted RNA was then used for cDNA synthesis using the TaqMan™ Advanced miRNA cDNA Synthesis kit and a SimpliAmp™ Thermal Cycler. The resulting amplified cDNA was diluted 1:10.

Individual mastermixes were prepared for each microRNA target, combining the specific miRNA assay, nuclease-free water, and TaqMan™ Fast Advanced Master Mix for qPCR. The mastermixes were distributed into a 96-well plate, followed by the addition of sample cDNA to corresponding wells. Each run included a negative control containing only nuclease-free water. Quantitative PCR was performed using the QuantStudio™ 3 Real-time PCR System.

### Statistical analysis

Statistical analysis was performed on qPCR data using Applied Biosystems qPCR analysis modules (Thermo Scientific™). The relative expression levels were calculated by subtracting the mean Ct value of the housekeeping microRNA-93 from the mean Ct values of the target microRNAs within each sample (ΔCt method).

While statistical methods are briefly outlined here, a comprehensive description of the statistical models and their theoretical background will be presented in a separate paper currently in preparation.

To ensure robust validation, the dataset was randomly split into training and testing subsets using an 80/20 ratio, resulting in 433 and 106 subjects, respectively.

For comparing clinical and lifestyle variables between groups, appropriate statistical tests were applied based on data distribution: Student's *t*-test for normally distributed metric variables, Mann–Whitney *U* test for non-normally distributed metric variables, and Chi-square test for categorical variables.

A multiple linear regression model was developed to estimate biological age, using chronological age as the reference. This age estimator will later be called miRNA-3Age. Model performance was evaluated using mean absolute error (MAE), the coefficient of determination (*R*^2^), and the median absolute error (MedAE).

To identify biological aging patterns, we calculated an age deviation index (BA-CA) as the difference between biological age (BA) and chronological age (CA). Negative values indicate a biologically younger profile, while positive values suggest accelerated aging. The relationships between the age estimator and various lifestyle and dietary characteristics were assessed using logistic regression, with odds ratios (OR) and 95% confidence intervals (CI) calculated to quantify the likelihood of being biologically younger.

All statistical analyses were performed using RStudio, with statistical significance set at *p* ≤ 0.05.

## Results

### Characteristics of the study population

For all participants gender, chronological age, biological age, the index between both, as well as the ΔCt values for miR-21, miR-24, miR-155 and BMI were evaluated (see Table 1). 539 adult participants were included in the analysis, mainly female. Neither chronological nor biological age demonstrated gender-specific differences.

**Table 1:** Demographic characteristics of the adult study cohort (*n* = 593).

**Variables**	**Study population**
Total	539
Gender [female]	346 (64%)
Age range [years]	18–81
Chronological age [years]	48.81 ± 13.02
Biological age [years]	48.78 ± 2.38
BA-CA [years]	0 ± 12.87
Biologically older	243 (45.1%)
miR-21 [ΔCt]	3.50 ± 0.74
miR-24 [ΔCt]	2.89 ± 0.56
miR-155 [ΔCt]	7.89 ± 0.77
BMI [kgm2]	24.73 ± 5.21

Data is expressed as mean with standard deviation; BA-CA, residual index, Biological age-chronological age.

Biological age was determined using parameters identified by *k*-fold cross-validation, with a standard deviation more consistent than chronological age.

As an index for the difference between chronological and biological age, BA-CA was calculated to determine the difference and to conclude if someone is biologically younger or older. The difference between biological and chronological age showed no significant gender influence.

### MiRNA correlating with chronological age

In Table 2 the results of Spearman rank test are listed. Analysis of the ΔCt values for miR-21 revealed a statistically significant negative association with chronological age.

**Table 2:** Correlation between ΔCt values of the miRNAs and chronological age (*n* = 539).

**miRNA**	**Correlation coefficient ρ**	***R* ^2^**	***p*-value**
miR-21	−0.12	0.020	**0.011**
miR-24	0.04	0.001	0.340
miR-155	−0.07	0.006	0.130

Spearman rank test was performed; *p*-values ≤ 0.05 are highlighted in boldface.

MiR-24 demonstrated a weak positive correlation with chronological age. The positive correlation coefficient ρ suggests that expression decreases with increasing age. The low *R*^2^ value and high *p*-value imply that this microRNA is not strongly associated with age variance and that the relationship with age is not statistically significant.

For the third microRNA, miR-155, a negative correlation with age was detected, indicating reduced expression over time. Similiar miR-24, miR-155 did not show statistical significance, and based on the *R*^2^ value, it could only explain less than 0.6% of the variance.

In summary, among the three microRNAs tested, only miR-21 demonstrated a statistically significant correlation with chronological age, although the *R*^2^ values were low for all three miRNAs.

### Prediction of age based on miRNA

To predict age despite the non-significant results for miR-24 and miR-155 with chronological age, a multivariate analysis was performed, as a combined model of these three miRNAs can still yield significant results (Table 3). Spearman rank test revealed that all three miRNAs are significantly associated with each other. The training sample set was used to develop this combined model with a 5-times repeated 10-fold cross-validation approach (see methods).

**Table 3:** Results of the linear model with performance metrics and predictive accuracy (*n* = 539).

**Predictors**	**Regression coefficient**	**95% CI**	***p*-value**
Intercept	66.73	[53.83, 79.63]	**0**
miR-21	−2.33	[−4.02, −0.64]	**0.007**
miR-24	2.90	[0.47, 5.46]	**0.020**
miR-155	−2.34	[−4.11, −0.54]	**0.011**
**Performance metrics**			
R^2^		0.051	
MAE [years]		10.4	
**Predictive accuracy**			
MedAE [years]		9	
Age correlation ρ		0.13 (*p* = 0.002)	

The linear model includes (chronological age – miR-21 + miR-24 + miR-155); MAE, mean absolute error; MedAE, median absolute error. Higher miRNA ΔCt values indicate lower expression levels and lead to a positive association between miRNA expression and chronological age. *P*-values ≤ 0.05 are highlighted in boldface.

A Regression Coefficient, 95% confidence interval as well as the *p*-value were determined.

With all variables known, an equation can be formulated where x_1_, x_2_, and x_3_ represent the ΔCt values of the microRNAs. Therefore, miR-21 was used as coefficient β1, miR-24 as coefficient β2 and miR-155 β3. The intercept was determined as β0.

Based on this equation, the miRNA-3Age span (Y) can be calculated, ranging from 40 to 60 years, which is considerably narrower than the chronological age range of 18 to 81 years (compare Table 1).

To assess the biological age model's performance, the mean absolute error (MAE) and *R*^2^ were evaluated. The MAE was determined to be 10.4 years, indicating that, on average, the predicted biological age deviates from the actual chronological age by approximately 10.4 years. The *R*^2^ value was 0.051, illustrating that 5.1% of the age variance refers to the predicting miRNAs' miR-21, miR-24, and miR-155.

Median absolute error (MedAE) and age correlation (correlation coefficient ρ) were used to assess predictive accuracy. As shown in Table 3, the MedAE was 9 years, indicating a significant median discrepancy of 9 years between miRNA-3Age and chronological age.

### Lifestyle and dietary characteristics influencing the biological age

The potential associations between lifestyle, dietary habits, and age acceleration were investigated using a logistic regression model.

#### Characteristics that decrease biological aging

Certain dietary and lifestyle factors were associated with a younger biological age relative to chronological age. Regular consumption of green tea, alcohol, and whole grain products, as well as moderate fish consumption and moderate levels of physical activity were linked to reduced biological aging. Among these, moderate fish consumption and frequent alcohol intake showed the strongest association. While daily whole grain consumption was beneficial, moderate intake did not appear to have a significant effect. Notably, engaging in physical activity one to two times per week was associated with a younger biological age, whereas higher frequencies of exercise did not demonstrate additional benefits.

**Figure 1:**
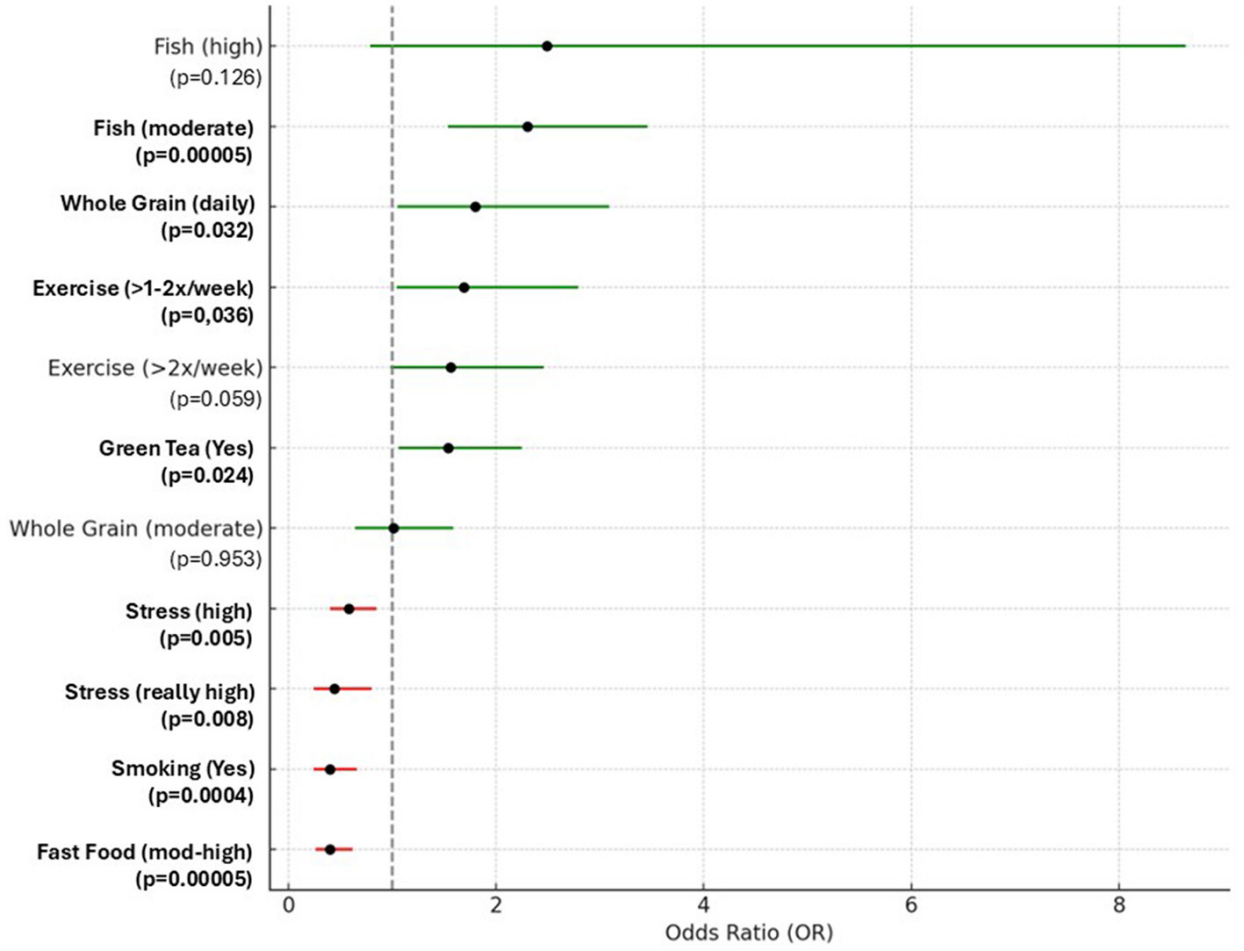
Lifestyle and dietary characteristics influencing biological age. The results in Figure 1 are presented as Odds Ratios (OR) and 95% Confidence Intervals (CI) with significant results highlighted in bold.

Figure 1 illustrates lifestyle and dietary characteristics that significantly influence biological age. Green color indicates factors associated with a reduction in biological age, while red represents those linked to an increase. The vertical line at OR = 1 serves as a reference point: values to the right indicate an increased relative likelihood of being biologically younger, and values to the left indicate a decreased likelihood.

#### Characteristics that increase biological aging

Smoking, frequent consumption of fast food, and high stress levels were among the main factors associated with an increased biological age (see Figure 1). Individuals who smoked or regularly consumed fast food were markedly less likely to be biologically younger than their chronological age. Likewise, elevated stress levels were linked to accelerated biological aging with more severe stress correlating with stronger negative effects.

#### Characteristics that do not influence biological aging

Analysis of certain dietary factors revealed no significant associations with biological aging. Fruit and vegetable intake did not show a significant relationship and demonstrated lower odds ratios in both moderate intake (OR = 1.24 [0.84, 1.83]) and high intake (OR = 0.68 [0.42, 1.09]).

Furthermore, dietary consumption patterns of milk and milk products, whether consumed seldom or every other day (OR = 1.43 [0.83, 2.47], *p* = 0.199) or daily (OR = 1.44 [0.83, 2.51], *p* = 0.193), and meat intake (moderate intake: OR = 1.01 [0.69, 1.49], *p* = 0.940; high intake: OR = 0.49 [0.22, 1.01], *p* = 0.055) were not associated with a biologically younger age.

Additionally, an increase in physical activity, compared to the lowest level, is associated with a higher likelihood of being biologically younger, with an 83% and 87% increase respectively. However, the results were not statistically significant (1 time per week: OR = 0.97 [0.32, 3.00], *p* = 0.951; 2–5 times per week: OR = 1.83 [0.68, 5.19], *p* = 0.235; 6–7 times per week: OR = 1.87 [0.69, 5.35], *p* = 0.223).

Similarly, consumption of sweets several times per week (OR = 0.83 [0.57, 1.20], *p* = 0.317), as well as liquid intake of 1–2 liters [OR = 1.52 [0.72, 3.28], *p* = 0.268] or over 2 liters (OR = 1.34 [0.94, 1.91], *p* = 0.109) did not influence biological aging. Lastly, frequent coffee consumption showed no significant effect on biological age (OR = 1.44 [0.97, 2.16], *p* = 0.074).

## Discussion

### Markers of aging

As numerous studies have demonstrated that miRNAs exhibit altered expression profiles with increasing age, they seem to be promising biomarkers for biological age assessment. The microRNAs selected for this investigation (miR-24, miR-21, and miR-155) were chosen based on their established roles in aging mechanisms, inflammatory pathways, and cellular senescence.

Our findings regarding miRNA expression corroborate previous research indicating a negative correlation between miR-24 and chronological age, as we observed decreased expression with increasing age (16, 26, 27, 33, 34). Furthermore, miR-21 exhibited upregulation with advancing age, which is consistent with findings from Olivieri et al. (22) and Mensà et al. (35).

For miR-155, the literature presents contradictory data, with some studies reporting age-associated upregulation (20), which aligns with our observations, while others suggest a more intricate relationship (26, 36). These discrepancies may be attributed to population sample variations, including differences in age distribution, lifestyle factors, and health status across studies, as well as methodological differences in extraction and quantification techniques. The variability in miR-155 expression patterns indicates that its relationship with aging is complex and may be influenced by multiple factors and therefore might change.

To estimate age, various analytical approaches can model temporal patterns in biological systems. We implemented linear regression analysis due to the significant interrelationships observed among all three miRNAs. Our regression analyses revealed negative correlations between miR-21, miR-155 ΔCt values and age, indicating upregulated expressing with increasing age, while miR-24 demonstrated downregulation.

The statistical significance of all three miRNAs in our linear model and the elevated *R*^2^ value support the hypothesis that these miRNAs' expression patterns are interdependent. This reinforces the concept that biological age estimation should incorporate multiple molecular markers rather than relying on individual biomarkers.

Our miRNA-3 Age model provides valuable insights into the development of accurate biological age estimation methods while highlighting the multifaceted and complexity of aging as a biological process.

### Nutrition and lifestyle

The question of human life expectancy continues to captivate both scientists and the general public. Twin studies suggest that genetic factors account for only about a quarter of an individual's lifespan, while the vast majority is shaped by diet, lifestyle and environmental influences (8). This underscores the importance of identifying and optimizing factors that are modifiable, such as daily habits, patterns, with the aim to enhance overall health and wellbeing (37).

#### Lifestyle influencing biological aging

##### Alcohol consumption

The relationship between alcohol consumption and health outcomes has attracted significant scientific attention. Alcohol consumption impacts cellular aging via oxidative stress, inflammation, affecting epigenetic and miRNA aging markers (38, 39).

Moderate alcohol consumption has been linked to several potential health benefits, including lower rates of morbidity, overall mortality, and coronary heart disease (CHD) mortality (40–42). These benefits are hypothesized to stem from alcohol's anti-inflammatory effects, which may reduce inflammatory markers such as IL-6 and C-reactive protein (CRP) (43).

The current study, along with previous research (43, 44), indicates a potential association between moderate alcohol intake and decelerated biological aging. Notably, individuals who consume alcohol daily are 2.85 times more likely to have a biological age lower than their chronological age compared to occasional drinkers. By contrast, individuals reporting heavy alcohol consumption showed epigenetic age acceleration of approximately 2.2 years relative to abstention, suggestion that dosage and pattern of drinking critically influence miRNA-based aging markers (37, 45).

It is important to consider that the younger biological age observed in daily drinkers may be more attributable to their overall lifestyle characteristics than to alcohol consumption *per se*. Most daily drinkers in this study were non-smokers, physically active, reported lower stress levels, and maintained a BMI under 25, factors that could potentially counteract the adverse effects of alcohol. Therefore, a comprehensive healthy lifestyle may exert a more substantial influence on biological aging than alcohol consumption in isolation.

Functionally, these three miRNAs modulate key inflammatory and stress-response pathways, highlighting their value as aging biomarkers. Clinically, integrating their profiling into the miRNA-3Age model could improve early detection of aging alterations, though broader health implications and confounders must be considered.

##### Stress

Psychological stress, especially when chronic, accelerated biological aging by persistently activating stress pathways, which induce oxidative damage, glucocorticoid imbalance, neuronal injury and telomere shortening, established biomarkers of accelerated biological aging (46, 47).

High psychological stress was significantly associated with accelerated biological aging and correlated changes in miR-21, miR-24, and miR-155. Individuals reporting high stress levels were 42% less likely to exhibit a biological age younger than their chronological age compared to low-stress controls, and those under extreme stress had a 56% reduction in odds, underscoring the impact of stress on miRNA-mediated aging pathways. These results align with Zannas et al., who linked chronic stress to epigenetic age acceleration, and suggest that cumulative lifetime stress may contribute up to 3.6 years of age difference (47, 48).

Therefore, these findings emphasize the need to investigate how different stress types, durations, and triggers affect miRNA aging pathways, and suggest that profiling miR-21, miR-24, and miR-155 can inform targeted stress-reduction strategies within anti-aging interventions.

##### Physical activity and exercise

Exercise is a intentionally planned, organized, repetitive form of physical activity aimed at improving physical fitness and health, such as running or weightlifting (48). In contrast, physical activity encompasses all movement, including leisure, transport, work, and household tasks. Both moderate and vigorous activities, such as walking, cycling, and sports, which can be performed at various skill levels, benefit and improve health across all ages (49). Importantly, while all exercise is physical activity, not all physical activity qualifies as exercise.

In this study, the effects of exercising 1–2 times per week were significantly associated with a 69% increased likelihood of having a biological age younger than chronological age compared to individuals who do not exercise regularly. Interestingly, no significant additional benefits were observed with exercise frequencies exceeding twice weekly, suggesting that engaging in higher frequencies of exercise may not further decelerate the biological aging process as measured by our miRNA-based biomarkers.

In contrast, physical activity levels were not significantly associated with biological age, despite trends suggesting an 83–87% higher likelihood of being biologically younger. This implies that structured moderate exercise may have a more pronounced effect on biological aging than general activity. Further research is needed to clarify these relationships and explore the molecular mechanisms underlying different forms of physical exertion. Further investigations are needed to clarify these associations and explore the potential underlying molecular mechanisms by which different forms of physical exertion may influence aging trajectories.

#### The effect of dietary factors on biological aging

Nutrition's impact on biological aging has become increasingly evident. The Mediterranean diet (MD), rich in omega-3 fatty acids, essential nutrients, and fiber, is linked to longevity and lower risks of mortality and cardiovascular disease (50).

Our findings support previous research linking specific Mediterranean diet components to reduced systemic inflammation, a key factor in aging. Daily whole grain intake was associated with a 1.8-fold higher likelihood of having a younger biological age, while moderate fish consumption showed a 2.3-fold increase, likely due to the anti-inflammatory effects of omega-3s (EPA and DHA) (43). This evidence strengthens the hypothesis that regular fish consumption may mitigate age-related pathologies. Notably, higher fish intake did not yield additional benefits, suggesting a potential threshold effect or confounding influences.

Contrary to prior research, we found no significant association between fruit and vegetable intake and reduced biological age. This finding contradicts previous research attributing anti-inflammatory and cardiometabolic benefits to these foods, which typically protect against chronic diseases including coronary heart disease, stroke, and type-2 diabetes (43). This unexpected result may reflect population-specific dietary patterns, produce quality, or unmeasured confounders. It's also possible that the miRNA biomarkers used lack sensitivity to the subtler effects of these foods.

Regular fast-food consumption was associated with a 60% lower likelihood of having a younger biological age, aligning with evidence linking diets high in refined carbs, saturated fats, and omega-6s to accelerated epigenetic aging. Poor nutritional quality likely accelerates biological aging through increased inflammation, oxidative stress, and sarcopenia, with emerging research also pointing to disrupted cellular function and lipid metabolism (51–53).

Our study reveals that regular green tea consumption is linked to a 54% higher likelihood of having a younger biological age, consistent with its known anti-inflammatory and antioxidant effects. These benefits are largely attributed to epigallocatechin-3-gallate (EGCG), a bioactive polyphenol with strong anti-senescent properties that mitigates oxidative stress and cellular aging (50, 54). Animal studies further suggest green tea may slow biological aging and extend lifespan, highlighting a potential causal role through enhanced cellular defense mechanisms (55). Future research should investigate optimal intake levels for maximal benefit.

Due to lifestyle and exposure differences across populations, our findings may not be universally generalizable. This highlights the need for tailored use of epigenetic markers in assessing biological age and evaluating interventions. Additionally, our investigation is subject to limitations including potential biases from self-reported lifestyle data and limited demographic diversity in our sample population.

This study offers valuable insights into the relationship between miRNA-3Age and lifestyle, supporting the notion that epigenetic age may be potentially reversible. The findings highlight the role of healthy diets in promoting longevity and suggest potential for personalized anti-aging strategies. Future research should examine longitudinal effects of lifestyle modifications on epigenetic age and explore the applicability of these results across different ethnic and genetic groups. Furthermore, investigating the relationship between epigenetic age and other established hallmarks of aging represents a promising direction for subsequent studies.


$$\begin{array}{c} y=66.73-2.33\cdot x1+2.96\cdot x2-2.34\cdot x3 \end{array}$$ (1)

## Data Availability

The original contributions presented in the study are included in the article/supplementary material, further inquiries can be directed to the corresponding author.
